# Assessing the clinical utility of measuring Insulin-like Growth Factor Binding Proteins in tissues and sera of melanoma patients

**DOI:** 10.1186/1479-5876-6-70

**Published:** 2008-11-24

**Authors:** Jessie Z Yu, Melanie A Warycha, Paul J Christos, Farbod Darvishian, Herman Yee, Hideko Kaminio, Russell S Berman, Richard L Shapiro, Michael T Buckley, Leonard F Liebes, Anna C Pavlick, David Polsky, Peter C Brooks, Iman Osman

**Affiliations:** 1 Departments of Dermatology, New York University School of Medicine, New York, NY, USA; 2 Division of Biostatistics and Epidemiology, Department of Public Health, Weill Medical College of Cornell University, New York, NY, USA; 3 Departments of Pathology, New York University School of Medicine, New York, NY, USA; 4 Departments of Surgery, New York University School of Medicine, New York, NY, USA; 5 Departments of Medicine, New York University School of Medicine, New York, NY, USA; 6 Maine Medical Center, Portland, Maine 04102, USA

## Abstract

**Background:**

Different Insulin-like Growth Factor Binding Proteins (IGFBPs) have been investigated as potential biomarkers in several types of tumors. In this study, we examined both IGFBP-3 and -4 levels in tissues and sera of melanoma patients representing different stages of melanoma progression.

**Methods:**

The study cohort consisted of 132 melanoma patients (primary, n = 72; metastatic, n = 60; 64 Male, 68 Female; Median Age = 56) prospectively enrolled in the New York University School of Medicine Interdisciplinary Melanoma Cooperative Group (NYU IMCG) between August 2002 and December 2006. We assessed tumor-expression and circulating sera levels of IGFBP-3 and -4 using immunohistochemistry and ELISA assays. Correlations with clinicopathologic parameters were examined using Wilcoxon rank-sum tests and Spearman-rank correlation coefficients.

**Results:**

Median IGFBP-4 tumor expression was significantly greater in primary versus metastatic patients (70% versus 10%, p = 0.01) A trend for greater median IGFBP-3 sera concentration was observed in metastatic versus primary patients (4.9 μg/ml vs. 3.4 μg/ml, respectively, p = 0.09). However, sera levels fell within a normal range for IGFBP-3. Neither IGFBP-3 nor -4 correlated with survival in this subset of patients.

**Conclusion:**

Decreased IGFBP-4 tumor expression might be a step in the progression from primary to metastatic melanoma. Our data lend support to a recently-described novel tumor suppressor role of secreting IGFBPs in melanoma. However, data do not support the clinical utility of measuring levels of IGFBP-3 and -4 in sera of melanoma patients.

## Background

Current therapeutic strategies focus on targeted drug development against pathways implicated in tumor signal transduction, cell cycle regulation, or immune response modulation. The insulin-like growth factor (IGF) axis is one such system which contributes to human malignancy, with overexpression of IGF1 receptor (IGF1R) noted in several cancers, including melanoma. The IGF system mediates signaling through a number of downstream pathways, including the RAS-RAF-mitogen-activated protein kinase (MAPK) and phosphatidylinositol 3-kinase (PI3K/AKT) pathways, with implications on the growth, proliferation, and survival of both normal and malignant cells. [1-3] Components of this system include the ligands IGF1 and IGF2, their cell surface tyrosine kinase receptors IGF1R and IGF2R, and seven IGF binding proteins (IGFBP).

IGF1R has been shown to play a role in a number of malignancies including melanoma, breast, prostate, and lung. [4-6] Therapeutic approaches which disrupt IGF1R signaling have been recently pursued, including receptor blockade through antisense oligonucleotides, monoclonal antibodies, or tyrosine kinase inhibitors. Several of these drugs are currently in Phase I trials as single agents or in combination with chemotherapy. [7-11] A critical aspect in the design of these trials has been the selection of appropriate surrogate end-points of treatment response. In addition to measuring objective tumor response, a few studies have incorporated serum measurement of IGFBP-3 as a biomarker of disease progression. [12-16] IGFBP-3, the most abundant IGFBP in circulation, is expressed in several cancers and was recently shown to exert IGF-independent inhibitory activity on angiogenesis *in vivo*.[17,18] IGFBP-3 has also been shown to be a p53-response gene that induces apoptosis in an IGF-independent manner. [19] Furthermore, recent data indicate IGFBP-3 may represent a potential node of cross-talk between DNA-damage and TGF-B1-dependent signaling pathways as it regulates several biomarkers of senescence [20]. Finally, combination therapy with retinoid X receptor-alpha ligands has led to synergistic induction of apoptosis in prostate cancer xenograft models.[21]

Few studies have reported on the expression of IGFBPs in melanoma.[22,23] DNA microarray analysis data have shown that IGFBP-3 expression is increased in metastases relative to primary tumors, with siRNA gene knockdown of IGFBP-3 in melanoma cells resulting in a reduction in cell motility, migration, and invasion.[23] Although these data support the role of IGFBP-3 as a potential biomarker in melanoma, serum concentrations were not measured, nor were clinicopathologic correlations or survival data presented.[23] In melanoma, IGFBP-7 has been shown to attenuate MAPK signaling, resulting in cellular senescence in BRAF mutant melanocytes and apoptosis in BRAF mutant melanoma cells, and data further suggest that it possesses potential tumor-suppressor activity.[24] IGFBP-4, the only member of the IGFBP family consistently shown to inhibit IGF activity, has also been examined for its role in cancer progression. Initial studies of IGFBP-4 gene therapy administered in colorectal cancer xenograft models resulted in a decrease in tumor micro-vessel counts and an increase in apoptosis.[25] Most recently, Zhu et al. have shown that IGFBP-4 has IGF-independent activity as a cardiogenic growth factor, and data suggest that it acts as a competitive inhibitor of the canonical Wnt signaling pathway [26]. IGFBP-4 levels may thus impact tumor angiogenesis and progression in colon cancer *in vivo*. To our knowledge, expression of IGFBP-4 in melanoma has not been previously reported.

In this study, we have assessed the clinicopathologic relevance of circulating as well as tumor-specific levels of IGFBP-3 and -4 in melanoma and aimed to define the most clinically relevant test to be integrated in correlative studies of clinical trials targeting IGF.

## Methods

### Study Population

The study cohort consisted of 132 melanoma patients (primary, n = 72; metastatic, n = 60; 64 Male, 68 Female; Median Age = 56) prospectively enrolled in the NYU IMCG between August 2002 and December 2006. Clinicopathologic, demographic, and survival data were recorded prospectively for all patients. The NYU Institutional Review Board approved this study and informed consent was obtained from all patients at the time of enrollment.

### Immunohistochemistry

IGFBP-3 protein expression was assessed by immunohistochemistry in formalin-fixed, paraffin embedded tissue specimens from 96 patients using mouse anti-human IGFBP-3 antibody (R&D Systems Minneapolis, MN), including 59 specimens from primary patients and 37 specimens from patients with metastatic disease. Similarly, formalin-fixed, paraffin embedded tissue specimens from 123 patients were examined using goat anti-human IGFBP-4 antibody (R&D Systems), including 66 specimens from primary patients, and 57 specimens from patients with metastatic disease. In brief, sections were deparaffinized in xylene (3 changes), rehydrated through graded alcohols (3 changes 100% ethanol, 3 changes 95% ethanol), and rinsed in distilled water. Heat-induced epitope retrieval was performed in 10 mM citrate buffer pH 6.0 in a 1200-Watt microwave oven at 90% power. IGFBP-4 was retrieved for 10 minutes and IGFBP-3 for 20 minutes, respectively. Sections were allowed to cool for 30 minutes and then rinsed in distilled water. Antibody incubations and detection were carried out at 37°C on a NEXes instrument (Ventana Medical Systems Tucson, Arizona) using Ventana's reagent buffer and detection kits, unless otherwise noted. Endogenous peroxidase activity was blocked with hydrogen peroxide. IGFBP-3 was diluted 1:50 and IGFBP-4 was diluted 1:25 and incubated overnight at room temperature. IGFBP-3 was detected by the application of a biotinylated goat anti-mouse (Ventana Medical Systems). IGFBP-4 was detected was detected using a biotinylated horse anti-goat (Vector Laboratories Burlingame, California) diluted 1:200 and incubated for 30 minutes. Both were followed by the application of streptavidin-horseradish-peroxidase conjugate. The complex was visualized with 3,3 diaminobenzidene and enhanced with copper sulfate. Slides were washed in distilled water, counterstained with hematoxylin, dehydrated and mounted with permanent media. Appropriate positive and negative controls were included with the study sections.

The expression of IGFBP-3 and -4 were scored by an attending pathologist (H.Y.), who was blinded to the patients' clinical data. Both IGFBP-3 and -4 protein expression were calculated based on the percentage of tumor cells which exhibited positive cytoplasmic staining. Immunoreactivity was assessed on a continuous scale with values ranging from undetectable levels (0%) to homogenous staining (100%) of invasive melanoma cells.

### Measuring IGFBP-3 and IGFBP-4 using ELISA

Serum specimens from 82 patients were collected and analyzed for IGFBP-3 (40 primary, 42 metastatic). IGFBP-4 expression was examined by ELISA assay in 80 of the 82 patients as the IGFBP-3 ELISA assay exhausted 2 patient sera samples. All serum samples were collected in 10 ml BD serum tubes, stored immediately at 4°C, and then centrifuged at 10°C for 10 minutes at 1,500–2,000 ×g. The supernatant serum was then aliquoted into 1.5 ml cryovials and stored at -80°C until further use.

Two commercially-available IGFBP-3 and -4 two-step sandwich ELISA assays were used to quantify the respective serum concentrations of these proteins (DSL-10-7300 Active IGFBP-3 ELISA and DSL-10-7300 Active IGFBP-4 ELISA, Diagnostic Systems Laboratories, Inc., Webster, TX). A 96-well flat bottom microtiter plate was coated with either mouse anti-human IGFBP-3 antibody or goat anti-IGFBP-4 antibody, respectively, and incubated for 1 hour at room temperature shaking at fast speed (500–700 rpm) on an orbital microplate shaker. After several washes with buffered saline containing a nonionic detergent (Wash Buffer), plates were incubated with either the anti-IGFBP-3 or the anti-IGFBP-4 antibody conjugated to the enzyme horseradish peroxidase in a protein-based (BSA) buffer with a non-mercury preservative (Antibody-Enzyme Conjugate Solution) for 30 minutes at room temperature while shaking at a fast speed (500–700 rpm). After three additional washes with Wash Buffer, 3,3',5,5'-tetramethylbenzidine in citrate buffer with hydrogen peroxide (TMB Chromogen Solution) was added to each well and incubated while shaking at room temperature for 10 minutes. The reaction was stopped with a (Stopping Solution) and the absorbance of the solution in the wells was read using a microplate reader set to 450 nm, and known concentrations of IGFBP-3 or -4 standards were utilized to establish a standard curve to extrapolate IGFBP-3 or -4 concentration within patient samples (DSL-10-7300 Active IGFBP-3 ELISA and DSL-10-7300 Active IGFBP-4 ELISA, Diagnostic Systems Laboratories, Inc., Webster, TX). Standards for IGFBP-3 were: Standard A, containing 0 ng/ml IGFBP-3 in a non-human serum with a non-mercury preservative, and IGFBP-3 Standard B-F, containing concentrations of respectively 5, 20, 40, 125, and 250 ng/ml IGFBP-3 in a non-human serum with a non-mercury preservative. The IGFBP-3 controls were two samples containing low and high concentrations of rhIGFBP-3 in a protein-based BSA buffer with a non-mercury preservative (10–6651 and 10–6652, Diagnostic Systems Laboratories, Inc., Webster, TX). Similarly, corresponding standards and controls for IGFBP-4 were used.

### Statistical Analysis

Baseline demographic and clinicopathologic characteristics were calculated for the study cohort. Associations between IGFBP-3 and -4 expression and age, gender, tumor thickness, histopathologic subtype, and metastatic tumor type were evaluated by the t-test (or Wilcoxon rank-sum test), the analysis of variance (ANOVA) test (or Kruskal-Wallis test), and the Spearman-rank correlation coefficient, as appropriate. For the analysis of histopathologic subtype, patients were collectively grouped into those who were diagnosed with superficial spreading melanoma, or "other" subtypes. To analyze mean and median IGFBP-3 and -4 tumor expression and sera concentrations, patients were grouped into those with primary disease and those with metastatic disease. The relationship between IGFBP-3 and -4 expression and patient overall survival was assessed with a hazard ratio derived from a Cox proportional hazards regression model. Overall survival was computed as the difference between the date of last follow-up and the date of initial diagnosis. Spearman correlation coefficients were used to examine the relationship between IGFBP-3 and -4 expressions in tissue specimens and concentration in sera. Sera data were represented by box and whisker plots, with upper and lower limits of the boxes indicating the 75^th ^and 25^th ^percentiles, respectively, and the central, horizontal line representing the median. Outliers are values that are more than 1.5 times the inter-quartile distance above the 75^th ^or below the 25^th ^percentile and are indicated by points outside of the box and whiskers. 39 patients had both sera and tumor specimens available for correlation of IGFBP-3 expression and 56 patients had both sera and tumor specimens available for IGFBP-4 correlation. All P values are two-sided with statistical significance evaluated at the 0.05 alpha level. All analyses were performed in SAS version 9.1 (SAS Institute, Inc., Cary, NC) and Stata version 8.0 (Stata Corporation, College Station, TX).

## Results

### IGFBP-3 and IGFBP-4 expression in primary melanomas

**Figure 1:**
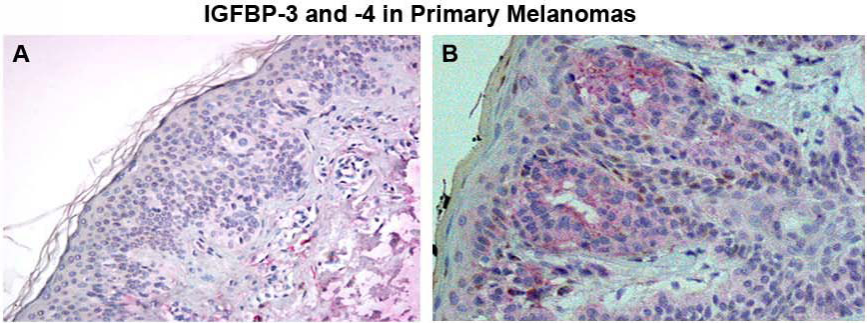
**IGFBP-3 and -4 expressions in primary melanoma tissue were evaluated with IHC**. (A) IGFBP-3 protein had low levels of expression in primary melanoma tissues, while (B) IGFBP-4 protein had high levels of expression in primary melanoma tissue. All images are at 20× magnification.

We examined the expression of IGFBP-3 and -4 in primary melanomas from 72 primary patients, according to 6^th ^Edition of the AJCC staging guidelines (Table 1). The median Breslow thickness for primary tumors was 0.45 mm, 40 tumors were axial, and 32 were located on the extremities. Histological examination revealed 68 superficial spreading type melanomas, and the remainder were nodular (n = 2) and lentigo maligna (n = 2) melanomas. Both IGFBP-3 and -4 exhibited cytoplasmic localization, but IGFBP-4 staining was more granular (Figure 1A and 1B). Median IGFBP-3 expression in primary melanoma tumor specimens was 80%, while median IGFBP-4 expression in primary tumors was 70%. Clinicopathologic correlation with IGFBP-3 and -4 expressions in primary melanoma samples revealed no significant association between IGFBP-3 or -4 tissue expression, or tumor thickness.

**Table 1:** Primary Patients Baseline Characteristics (n = 72)

**Variables**	***n(%)***
**Age (y)**	
Mean (± SD)	55.1 ± 16.3
Median	54.0
**Sex**	
Male	30 (41.7)
Female	42 (58.3)
**Stage**	
I	72 (100)
II	0
**Thickness (mm)**	
Mean (± SD)	0.50 ± 0.25
Median	0.45
**Histologic Type**	
Superficial Spreading	68 (94.4)
Nodular	2 (2.78)
Lentigo Maligna Melanoma	1 (1.39)
**Anatomic Location**	
Axial	40 (55.6)
Extremity	32

### IGFBP-3 and IGFBP-4 expression in metastatic melanomas

**Figure 2:**
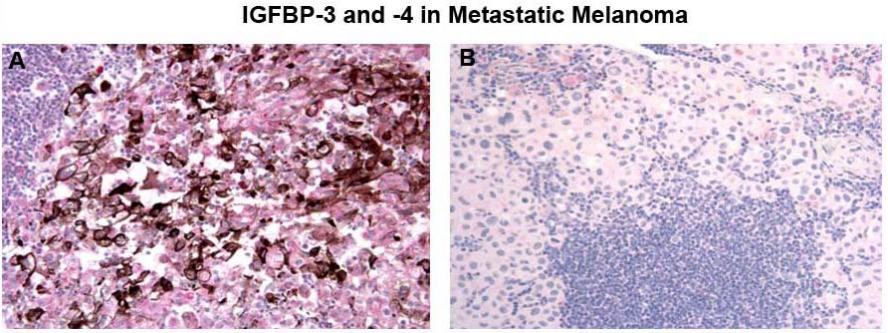
**IGFBP-3 and -4 expressions in metastatic melanoma tissue were evaluated with IHC**. (A) IGFBP-3 protein had high levels of expression in metastatic melanoma tissue. (B) IGFBP-4 low levels of expression in metastatic melanoma tissue. All images are at 20× magnification.

We examined the expression of IGFBP-3 and -4 in 60 melanoma tumors from 60 patients with metastatic disease according to 6^th ^Edition of the AJCC staging guidelines (Table 2). Again, both IGFBP-3 and -4 exhibited cytoplasmic localization (Figure 2A and 2B). The median IGFBP-3 expression in metastatic melanoma specimens was 90%, slightly higher than its expression in primary tumors. The median IGFBP-4 expression in metastatic melanoma specimens was 10%, significantly lower than IGFBP-4 expression in primary tumors (p = 0.01, Wilcoxon rank-sum test). Clinicopathologic correlation with IGFBP-3 and -4 expressions in metastatic melanoma samples revealed no significant association between IGFBP-3 or -4 expression and gender, regional versus distant disease, and presence of multiple metastases. While neither IGFBP-3 or -4 tissue or sera expression had any significant correlation with overall survival, we did observe a trend towards shorter median survival in patients with elevated IGFBP-4 tissue expression (mean = 32.7%) compared to those with lower IGFBP-4 expression (mean 24.9%, p = 0.07).

**Table 2:** Metastatic Patients Baseline Characteristics (n = 60)

**Variables**	***n(%)***
**Age (y)**	
Mean (± SD)	59.8 ± 17.0
Median	61.0
**Sex**	
Male	26 (43.3)
Female	34 (56.7)
**Stage**	
III	34 (56.7)
IV	26 (43.3)
**Presence of Multiple Metastases**	
Yes	26 (43.3)
No	34 (56.7)
**Anatomic Location**	
Regional Skin/Subcutaneous	20 (33.3)
Regional Lymph Node	26 (43.3)
Distant Lymph Node	2 (3.33)
Distant Skin/Subcutaneous	6 (10.0)
Visceral	6 (10.0)

### Association between IGFBP-3 expression in tissue and sera

**Figure 3:**
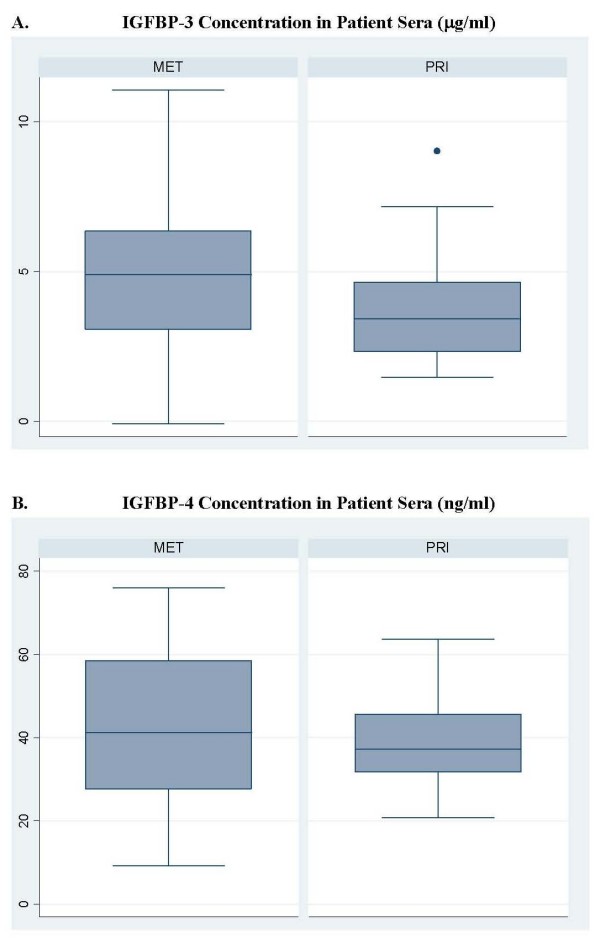
**IGFBP-3 and -4 sera concentration for primary and metastatic patients**. A. Median IGFBP-3 in sera of primary patients was 3.4 μg/ml compared with 4.9 μg/ml, in metastatic patients (p = 0.08 by Wilcoxon rank-sum test). B. Median IGFBP-4 in sera of primary patients was 37.2 ng/ml compared with 41.2 ng/ml, in metastatic patients (p = 0.25 by Wilcoxon rank-sum test). The boxes represent the inter-quartile distances with upper and lower limits of the boxes indicating the 75^th ^and 25^th ^percentiles, respectively, and the central, horizontal line representing the median. Outliers are values that are more than 1.5 times the inter-quartile distance above the 75^th ^or below the 25^th ^percentile and are indicated by points outside of the box and whiskers.

Of the 82 sera samples analyzed by the IGFBP-3 ELISA assay, 20 were eliminated from analysis due to hemolysis and of the 62 remaining samples, 27 were from primary patients and 35 were from metastatic patients. A trend for greater median IGFBP-3 sera concentration was observed in metastatic versus primary patients (4.9 ug/ml vs. 3.4 ug/ml, respectively, p = 0.26, Wilcoxon rank-sum test, Figure 3A). Data regarding both tissue and sera IGFBP-3 expression was available for 39 patients. No correlation was observed between IGFBP-3 sera concentration and tissue expression (p = 0.25). IGFBP-3 sera concentration did not correlate significantly with gender, age, thickness, anatomic location, regional versus distant disease, or presence of multiple metastases (data not shown).

### Association between IGFBP-4 expression in tissue and sera

IGFBP-4 expression was examined by ELISA assay in 80 of the 82 patients as the IGFBP-3 ELISA assay exhausted 2 patient sera samples. Of the 80 samples, 20 were eliminated from analysis due to hemolysis observed in those aliquots. IGFBP-4 serum levels were quantified in 60 viable samples (26 primary, 34 metastatic), and no significant difference was observed between the median IGFBP-4 concentration in primary patients versus metastatic patients (37.2 ng/μl vs. 41.42 ng/μl, respectively, p = 0.25, Wilcoxon rank-sum test, Figure 3B). Data regarding both tissue and sera IGFBP-4 expression was available for 56 patients. Analyses revealed no association between the expression of IGFBP-4 in tissue and its concentration in sera (p = 0.57). There was no association between IGFBP-4 sera concentration and gender, thickness, anatomic location, regional versus distant disease, and presence of multiple metastases.

## Discussion

Our study documents several important observations. First, we demonstrate that tissue expression of IGFBP-4 decreases in the progression from primary to metastatic melanoma. Furthermore, we did not detect a correlation between sera concentration and tissue expression for either IGFBP-3 or -4. These data suggest that IGFBPs localized to the tumor compartment may be differentially regulated compared to circulating IGFBPs. We also show that tissue expression of IGFBP-3 and -4 may be more clinically relevant than circulating levels, results which could reflect their systemic proteolytic cleavage and physiologic regulation by other endocrine hormones. While our study did not use semiquantitative analysis to score the immunoreactivity of the specimens, future studies incorporating this analysis will generate immunoreactivity data that may more closely reflect relative gene product expression levels in tissue. Thus, it is possible that mechanisms by which IGFBPs are produced and/or degraded differ between the tumor microenvironment and plasma, leading to increases in tumor expression without concurrent increases in circulatory levels, or vice versa.

We report for the first time data which demonstrate the up-regulation of IGFBP-4 expression in primary versus metastatic melanoma specimens, and these data suggest that IGFBP-4 may function as a tumor suppressor. This is consistent with its biologic function as an inhibitor of IGF activity.[27] While previous studies investigating the overexpression of IGFBP-4 in both colorectal and prostate cancers *in vivo *found evidence of decreased tumor proliferation, these correlations have not yet been performed in melanoma.[25,28] In this regard, our group has found evidence to suggest that integrin αvβ3 can mediate the expression of IGFBP-4 Specifically, treatment of M21 melanoma cells with a monoclonal antibody directed against αvβ3 results in an elevation of IGFBP-4 levels both *in vitro *and *in vivo*. Furthermore, immunohistochemistry data from 132 melanoma patient tumor specimens (primary, n = 72; metastatic, n = 63) demonstrate that in the progression from primary to metastatic melanoma, IGFBP-4 expression decreases while integrin avb3 expression increases (data not shown). These findings further support the potential role of IGFBP-4 as an endogenous inhibitor of angiogenesis and tumor growth in melanoma.

IGFBPs have been shown to have IGF-independent activities in multiple cellular pathways. [19,20,26] Wajapeyee *et al *recently described a novel function of IGFBPs in BRAF-mediated cellular senescence.[24] Specifically, data suggest that IGFBP-7 acts through a negative feedback loop to attenuate MAPK signaling, resulting in cellular senescence in BRAF mutant melanocytes and apoptosis in BRAF mutant melanoma cells. Furthermore, they found a high level of IGFBP-7 expression in BRAF mutant nevi and undetectable levels in BRAF mutant melanomas, suggesting that this protein may act as a tumor-suppressor in melanoma. Although IGFBP-3 and -4 have not been examined in this context, it is possible that other IGFBPs have implications on BRAF signaling and could potentially serve as surrogate markers for BRAF positivity. Further examination of IGFBPs in relation to MAPK signaling and BRAF mutation status are thus warranted.

Our data on IGFBP-3 expression in melanoma do not strongly support a previously published report which found up-regulation of IGFBP-3 in melanoma metastases compared to primary melanoma specimens.[23] Our data indicates only a slight difference in IGFBP-3 expression between metastatic and primary tumors, and there was no significant difference in IGFBP-3 sera levels between metastatic and primary patients. In fact, IGFBP-3 sera levels of the majority of the melanoma patients fell within the normal expected range for adults. Interestingly, these data also contrast with what has been recently presented in prostate cancer. In those studies, IGFBP-3 was shown to exert direct, tumor-suppressive effects via IGF-independent inhibition of angiogenesis[18] and both IGF-dependent and -independent induction of apoptosis. [29-31] Thus, it appears that IGFBP-3 plays different roles among different cancers.

The prognostic relevance of IGFBP-3 or -4 expressions in melanoma also requires further investigation. It has been previously reported that low tumor expression of IGFBP-3 in patients with primary hepatocellular carcinoma was independently associated with poor survival.[32] Consistent with these data, high plasma levels of IGFBP-3 were shown to be predictive of longer progression-free survival in patients with advanced non-small cell lung cancer.[33] However, to our knowledge, no reports exist on the prognostic relevance of IGFBP-3 or -4 expressions in melanoma.

Our data do not support the further development of IGFBPs as surrogate endpoint biomarkers for treatments targeting IGF1R. Although sera shedding of IGFBP-3 increased slightly in the progression from primary to metastatic melanoma, the majority of IGFBP-3 sera levels in the melanoma patient cohort fell within the expected range for healthy adults (1.5–5.6 ug/ml). Standard IGFBP-4 sera levels have yet to be established for comparison (Diagnostic Systems Laboratories, Inc.). Interestingly, nearly 30% of patients studied (10 primary, 15 metastatic patients) had IGFBP-3 sera concentrations up to twice the expected normal maximum, and 5 of the 15 metastatic patients with high serum IGFBP-3 died of melanoma less than 2 years after the date of blood collection. While in principle, taking multiple sera collection points may be more informative, this is an observation made from a small subset of the patients studied. Data from the study at large indicate that the majority of patients' IGFBP-3 sera levels fell within the expected normal range for adults. Furthermore, it is known that circulating levels of IGFBP-3 and -4 can be affected by multiple, systemic confounding factors, including diet, exercise, pregnancy, growth hormone, and age.[34,35] Therefore, multiple collections will not change the overall conclusion that there is no significant difference in sera levels of either IGFBP-3 or -4 between primary and metastatic melanoma patients.

## Conclusion

These data indicate that decreased IGFBP-4 tumor expression might be a step in the progression from primary to metastatic melanoma. Furthermore, our data lend support to a recently-described novel tumor suppressor role of secreting IGFBPs in melanoma. However, data do not support the clinical utility of measuring levels of IGFBP-3 and -4 in sera of melanoma patients.

## Abbreviations

(IGF): Insulin-like Growth Factor; (IGF1R): Insulin-like Growth Factor-1 receptor; (IGFBP-3): Insulin-like Growth Factor Binding Protein-3; (IGFBP-4): Insulin-like Growth Factor Binding; Protein-4; (MAPK): Mitogen-activated protein kinase; (IMCG): Interdisciplinary Melanoma Cooperative Group; (PI3K/AKT): Phosphatidylinositol 3-kinase.

## Competing interests

The authors declare that they have no competing interests.

## Authors' contributions

JZY made contributions to the study design, acquisition of data, analysis and interpretation of data, and the writing of this manuscript. MAW participated in the analysis and interpretation of data and the writing of this manuscript. PJC performed all statistical analyses. FD and HK reviewed all specimens for clinicopathological data and tumor content. HY scored all slides after immunohistochemistry to evaluate IGFBP expression in tumor. RSB, RLS, and ACP enrolled all patients into the IMCG and assisted in the conception of this study. MTB performed ELISA assays and assisted in the writing of this manuscript. LFL assisted in the conception, design, and coordination of this study. DP contributed to data analysis and writing of this manuscript. PCB provided pre-clinical data supporting this study, and he assisted in the conception, design, and writing of this manuscript. IO conceived this study, oversaw its design and coordination, supervised the analysis and interpretation of the data, and writing the manuscript. All authors read and approved the final manuscript.
